# A comparison of hemagglutination inhibition and neutralization assays for characterizing immunity to seasonal influenza A

**DOI:** 10.1111/irv.12408

**Published:** 2016-08-27

**Authors:** Shaun Truelove, Huachen Zhu, Justin Lessler, Steven Riley, Jonathan M. Read, Shuying Wang, Kin On Kwok, Yi Guan, Chao Qiang Jiang, Derek A. T. Cummings

**Affiliations:** ^1^ Department of EpidemiologyJohns Hopkins Bloomberg School of Public HealthBaltimoreMDUSA; ^2^ State Key Laboratory of Emerging Infectious DiseasesThe University of Hong KongHong Kong SARChina; ^3^ WHO Collaborating Centre for Infectious Disease Epidemiology and ControlSchool of Public HealthLi Ka Shing Faculty of MedicineThe University of Hong KongHong Kong SARChina; ^4^ School of Public HealthImperial CollegeLondonUK; ^5^ Department of Epidemiology and Public HealthInstitute of Infection and Global HealthUniversity of LiverpoolNestonUK; ^6^ Guangzhou No. 12 HospitalGuangzhouGuangdongChina; ^7^ Department of BiologyUniversity of FloridaGainesvilleFLUSA; ^8^ Emerging Pathogens InstituteUniversity of FloridaGainesvilleFLUSA

**Keywords:** cross‐protection, hemagglutination inhibition test, immunity, influenza, microneutralization test, neutralization test

## Abstract

**Background:**

Serum antibody to influenza can be used to identify past exposure and measure current immune status. The two most common methods for measuring this are the hemagglutination inhibition assay (HI) and the viral neutralization assay (NT), which have not been systematically compared for a large number of influenza viruses.

**Methods:**

A total of 151 study participants from near Guangzhou, China, were enrolled in 2009 and provided serum. HI and NT assays were performed for 12 historic and recently circulating strains of seasonal influenza A. We compared titers using Spearman correlation and fit models to predict NT using HI results.

**Results:**

We observed high positive mean correlation between HI and NT assays (Spearman's rank correlation, ρ=.86) across all strains. Correlation was highest within subtypes and within close proximity in time. Overall, an HI=20 corresponded to NT=10, and HI=40 corresponded to NT=20. Linear regression of log(NT) on log(HI) was statistically significant, with age modifying this relationship. Strain‐specific area under a curve (AUC) indicated good accuracy (>80%) for predicting NT with HI.

**Conclusions:**

While we found high overall correspondence of titers between NT and HI assays for seasonal influenza A, no exact equivalence between assays could be determined. This was further complicated by correspondence between titers changing with age. These findings support generalized comparison of results between assays and give further support for use of the hemagglutination inhibition assay over the more resource intensive viral neutralization assay for seasonal influenza A, although attention should be given to the effect of age on these assays.

## 1 Introduction

Accurate measurement of individuals' pathogen exposure history is an essential tool for understanding risk factors of infection and population‐scale patterns of transmission. Determined through a variety of methods, the concentration of antibodies in sera is considered the gold standard method to estimate past exposure to pathogens. Two of the most common methods for measuring serum antibody to influenza are the hemagglutination inhibition (HI) and virus neutralization (NT) assays.^1^ Although both tests serve as measures of antibody concentration in sera, they have important differences in how they are conducted and how they measure immunity. The HI test, which is fast and relatively easy to perform, is considered to be easily standardized and reproducible across laboratories. However, only the effect of antibodies on the hemagglutination process, by which a virus binds to red blood cells, is measured with HI, and the endpoint is only a correlate of the ability of antibodies to inhibit virus infection of host cells.^2^, ^3^ In contrast, NT assays, also known as microneutralization assays, measure the titer needed to block the cytopathic effects of the virus, by measuring antibodies that block entry of the virus into the cell, internalization of the virus, and fusion of the HA. Although NT is intuitively more appealing because it more closely mirrors the disease process in vivo, it is more time‐consuming and expensive and considered harder to standardize across laboratories.^2^, ^3^

Despite the widespread usage of these two methods, there have been few formal comparative studies of these measures. In a 2007 study by Stephenson et al., HI and NT tests were performed in 11 laboratories to investigate reproducibility of each assay for detection of anti‐H3N2 influenza antibodies. They found significantly higher variation in NT results between laboratories than in HI results, yet better discrimination among NT and generally limited correlation between the tests.^2^ In a follow‐up study of anti‐H1N1pdm antibodies, significant correlation between HI and NT was found, yet the conversion factors between laboratories varied significantly. Furthermore, NT titers were both significantly higher and significantly more variable than HI titers.^3^

The difference in reliability between laboratories with these two assays is a direct result of how they are measured. Hemagglutination inhibition and viral neutralization assays assess the level of functional immunity to a virus in a similar manner, both using serial dilution of sera applied to a fixed amount of virus to determine at which titer of sera the virus is effectively inhibited. The difference is in the biological mechanism used as an indicator for inhibition. The HI assay utilizes the natural process of viral hemagglutination, a process in which a lattice forms by binding of viruses to red blood cells; this process is blocked when sufficient antibody with affinity to the virus is present. A serum HI titer of ≥40 is assumed to indicate a 50% reduction in susceptibility compared with an individual with undetectable titer.^4^, ^5^, ^6^ The NT assay, in contrast, measures cytopathic effects of the virus, the invading and killing of cells, through plaque formation. Again, the antibodies in the sample serum are tested for their ability to block this activity. Results are expressed as reciprocal of the highest dilution at which virus infection is blocked.^7^

The viral neutralization test is valued for its high sensitivity and specificity, which have been found to be higher than for microneutralization fluorescent antibody test (MFA) and HI, although some have indicated similar sensitivity and specificity between HI and NT tests for certain viruses, including influenza A H1N1 2009.^2^, ^5^, ^8^, ^9^ Additionally, it has been found to be more strain‐specific than HI for seasonal and H5N1 viruses, and HI tests have been found to be insensitive for the detection of human antibody responses to avian hemagglutinin, especially when intact virus is present.^2^, ^6^ According to Gross and Davis, the neutralization test “appears to detect lower levels of viral antibody than does the HI test,” a difference that “may be related to the high serum concentrations and the additional viral antigens detected by NT.”^10^ It is, however, a laborious and time‐consuming procedure, making it less suitable for testing large numbers of samples.^11^ Disadvantages of NT include its difficulty level and time required to perform, the need for live virus, and that technical aspects of the assay can affect titers.^3^, ^8^ However, the major disadvantage of it has been poor reproducibility between laboratories.^2^, ^3^

In addition to low sensitivity, in particular as compared with radioimmunoassay (RIA) and enzyme‐linked immunosorbent assay (ELISA), disadvantages of HI include subjectivity of result interpretation and reliability issues in relation to freshness of reagents. For both tests, immunity measurement is not exact, but rather based on titer cut points, and the endpoint of both assays requires visual inspection. Other than cost, ease, and reduced variability, a major advantage of HI over NT for measurement of seasonal influenza immunity is that HI does not require cytopathy, which does not always occur for each influenza virus in this assay.^7^

Hemagglutination inhibition and NT assays have been utilized for years to investigate influenza immunity, although only a few studies have directly compared the assays' influenza antibody detection capabilities, and most of these studies evaluated vaccine‐derived immunity.^2^, ^3^, ^12^ Here, we compare HI and NT antibody titers from a sample of individuals from Guangdong Province, China, in order to formally compare the performance of HI and NT titers for measuring naturally derived immunity to twelve historic and recently circulating strains of influenza A. These twelve strains are seasonal influenza strains of both H3N2 and H1N1 subtypes that have been or are in broad circulation since 1968. Additionally, we will attempt to determine an equivalence factor between HI and NT titers for direct translation and comparison of results from both assays.

## 2 Methods

### 2.1 Sample collection

Sera samples were collected from 151 study participants from randomly selected households in five study locations in a transect extending to the northeast of Guangzhou, China, from 8 July 2009 to 21 September 2009, as described in Lessler et al.^13^ All study participants were administered informed consent, and a single blood sample was collected in a 5‐mL non‐heparin containing vacuum tube from each. Sera were extracted and split at Guangzhou Hospital, and testing and storage were done at Shantou University.

### 2.2 Laboratory testing

Hemagglutination inhibition (HI) and neutralization (NT) assays were performed for twelve historic and recently circulating strains of influenza A: nine H3N2 strains (A/Hong Kong/1/1968, A/Victoria/3/1975, A/Bangkok/1/1979, A/Beijing/353/1989, A/Wuhan/359/1995, A/Fujian/411/2002, A/Shantou/90/2003, A/Shantou/806/2005, and A/Shantou/904/2008), two previous seasonal H1N1 strains (A/Shantou/104/2005 and A/Shantou/92/2009), and one 2009 pandemic H1N1 strain (A/California/07/2009). Laboratory tests were performed as described in Lessler et al.^13^ In brief, the 50% tissue culture infectious dose (TCID_50_) for each virus was determined on Madin‐Darby canine kidney (MDCK) cells. The neutralization concentration was considered to be the reciprocal of the highest dilution of sera at which 50% of wells were infected (calculated using the method of Reed and Muench).^14^ Sera were thawed, treated with a receptor‐destroying enzyme (RDE) to remove non‐specific inhibitors, then heat‐inactivated at 56°C for 30 minutes. RDE‐pre‐treated sera were absorbed with Turkey red blood cells before being used in HI or NT tests, to remove substances causing non‐specific agglutination. Antibody titer was determined by testing serial twofold dilutions from 1:10 to 1:1280 in duplicate, resolving uncertain results by additional quadruplicate tests. Positive and negative control sera were also tested. HI assay was conducted with 0.5% turkey erythrocytes using four hemagglutination units. NT tests were carried out by mixing serially diluted sera with 100 TCID_50_ of MDCK cell‐adapted viruses of each strain, incubated for 1 hour at 37°C, and added to a MDCK cell monolayer. Cytopathic effect was read and hemagglutination assays performed to detect the presence of viral replication 3 days after inoculation. The highest dilution with complete protection of the cell monolayer in >2 quadruplicate wells was considered to be the NT titer. Both assays were performed in the same laboratory within the same week of each of other by an overlapping study team. Samples undergoing each assay were handled and processed identically, undergoing same sequence of freezing and thawing before testing. The full protocol is described in the WHO Manual on Animal Influenza Diagnosis and Surveillance.^15^

### 2.3 Statistical analysis

Hemagglutination inhibition and NT titers were compared using Spearman correlation between log2(HI/5) and log2(NT/5) titers on the 12 reference strains. We refer to titer values as log(HI) and log(NT) for the remainder of this manuscript. Cross‐correlation between strains was calculated by the same method.

We examined the ability of one assay to predict status based on the other. We treated detectable NT titers as the gold standard (i.e., NT titer ≥20) to calculate receiver operator characteristic (ROC) curves using different cutoffs for “positivity” for equivalent HI titers to predict status. Linear regression models were fit to predict log(NT) titer using log(HI) titer. All statistical analyses were conducted in r Statistical Software, version 3.0 (www.cran.org).

## 3 Results

One hundred and fifty‐one study participants were included in this analysis. The median age among them was 45 years (SD=18.75; range=7–81), with 7.3% aged <15 years (11/151). In total, 54.3% of participants were male (82/151). Among all participants, 66.2% (100/151) had never received an influenza vaccination, with only 7 (4.7%) receiving vaccines during the current or previous year, 6 (4.0%) within the last 2‐5 years, and 19 (12.6%) >5 years prior; 19 (12.6%) were unsure or unknown.

**Figure 1:**
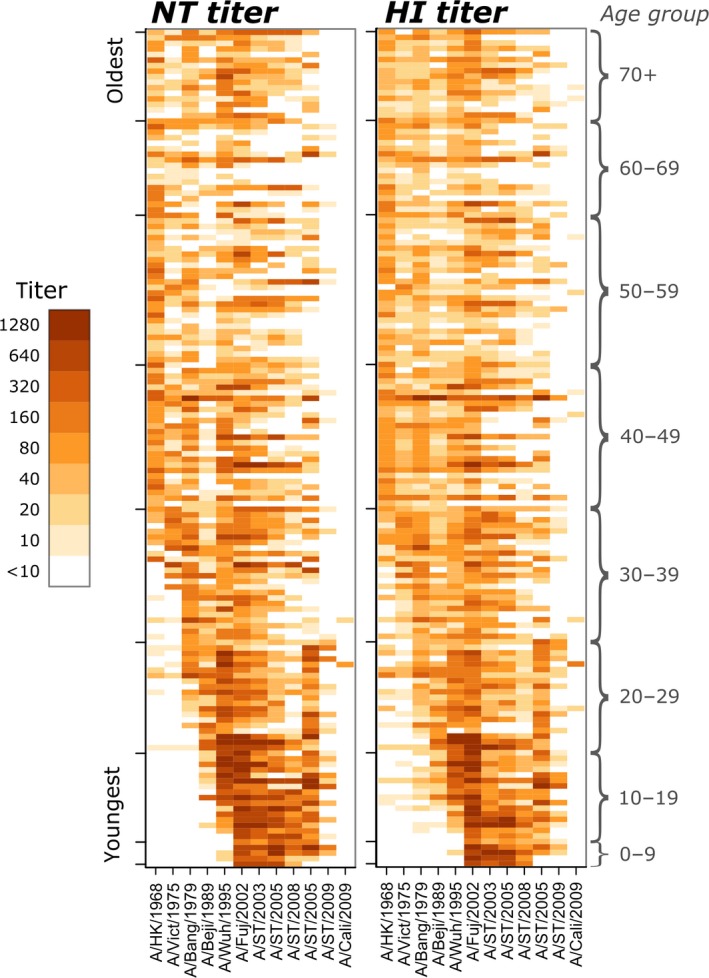
Neutralization assay (NT) (left) and hemagglutination inhibition assay (HI) (right) titers for each of the 151 participants in the study plotted by rank of age (oldest at top). Color indicates the titer measured by each assay. Strains are indicated on the *x*‐axis of each figure

**Figure 2:**
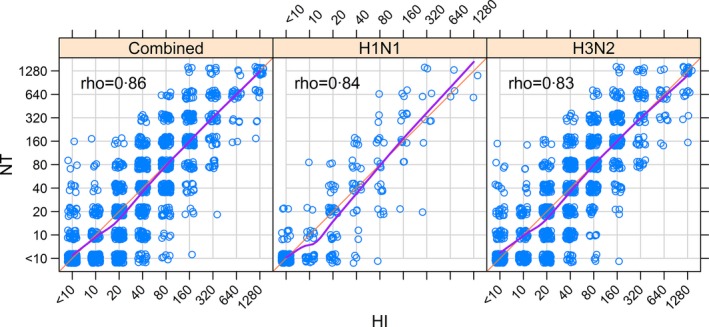
Correlation of HI and NT titers for all influenza A strains, H3N2 strains, and H1N1 strains. The orange lines indicate perfect correlation between HI and NT titers. The purple line represents the overall smoothed mean of the data

**Figure 3:**
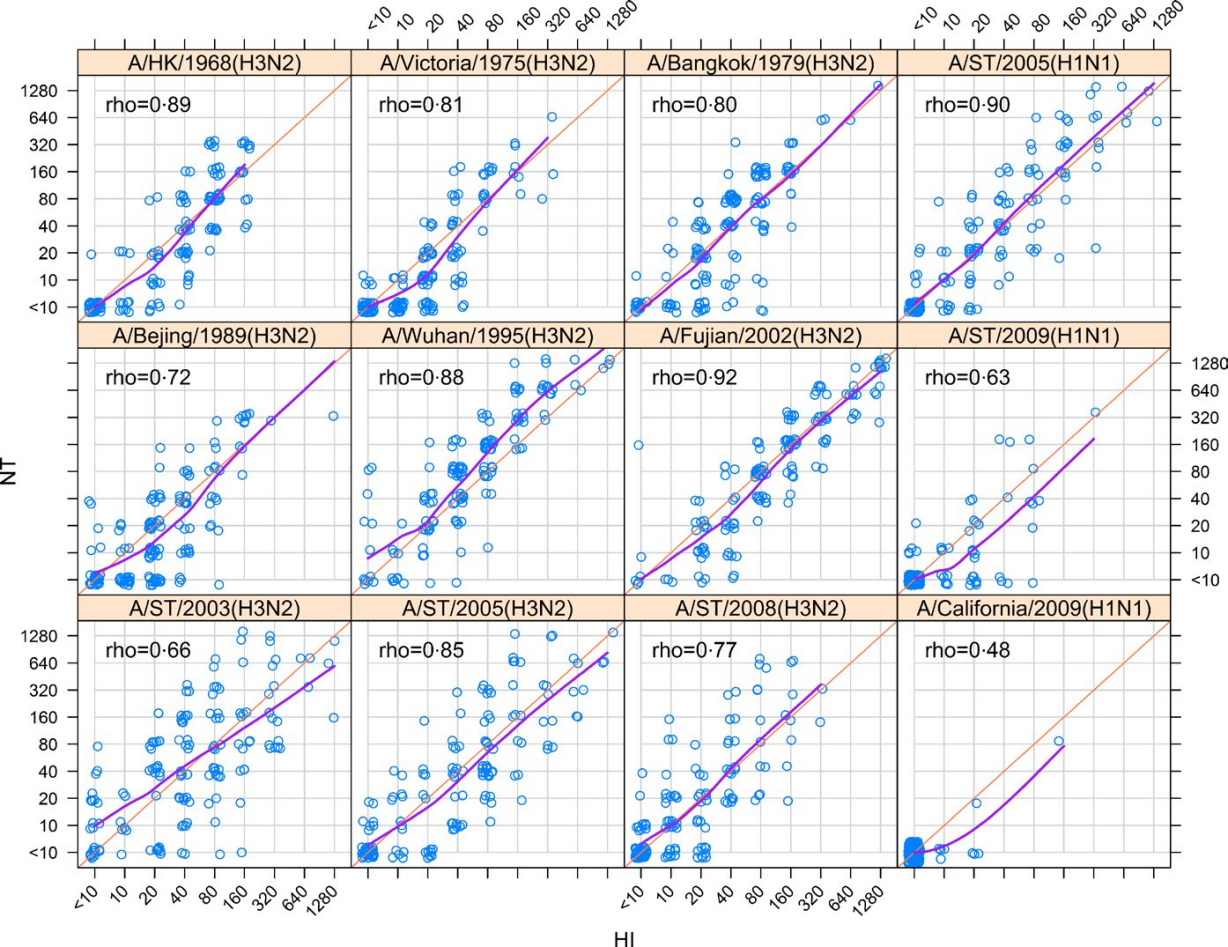
Correlations of HI and NT titers by influenza A strain. The orange lines indicate perfect correlation between HI and NT titers. The purple line represents the overall smoothed mean of the data

Antigenic testing of sera from the 151 pilot study participants for twelve strains of influenza A virus (shown in Fig. 1) indicated a high positive mean correlation between hemagglutination inhibition and neutralization assays (Spearman's rank correlation, ρ=.86), and both H3N2‐ and H1N1‐specific correlations were found to be high (ρ=.84; ρ=.83) (Fig. 2). Strain‐specific correlation varied by year and serotype, with the highest correlation observed for A/Fujian/411/2002 (H3N2) (ρ=.92) and the lowest observed for A/California/07/2009 (H1N1; ρ=.48; Fig. 3). Two strains of H1N1, A/Shantou/92/2009 and A/California/07/2009, had very low titer results overall (Table 1), resulting in low correlation coefficients. For A/Shantou/92/2009, however, HI titers appear to be more discriminating at the lower end, which indicates that the HI assay might be more sensitive for this strain than NT (Fig. 3). This relationship appears to be common across several strains examined (Fig. 3). Correlation of titers from different influenza strains using the same assay was highest between strains of the same subtype and in close proximity in time (Fig. S2). The highest correlations were found between A/Shantou/806/2005 and A/Shantou/90/2003 (ρ=.83) and A/Shantou/806/2005 and A/Shantou/904/2008 (ρ=.82) for HI, and between A/Fujian/411/2002 and A/Shantou/90/2003 (ρ=.88) for NT.

**Table 1:** Hemagglutination inhibition and NT median titers and proportions of titers equal to or greater than the current gold standards of both for all twelve recently circulating influenza strains

	HI titers		NT titers	
Strain	Median titer	Proportion titers ≥40 (%)	Median titer	Proportion titer ≥20 (%)
A/Hong Kong/1/1968	40	55.00	20	59.60
A/Victoria/3/1975	40	31.10	10	36.40
A/Bangkok/1/1979	40	56.30	40	67.50
A/Beijing/353/1989	20	35.80	10	47.70
A/Wuhan/359/1995	40	69.50	80	84.80
A/Fujian/411/2002	80	80.80	80	83.40
A/Shantou/90/2003	40	62.30	40	75.50
A/Shantou/806/2005	40	57.00	40	60.30
A/Shantou/904/2008	10	28.50	10	45.00
A/Shantou/104/2005	20	42.40	20	55.00
A/Shantou/92/2009	<10	9.30	<10	11.30
A/California/07/2009	<10	0.70	<10	1.30
Total	—	44.00	—	52.30

Linear regression of log(NT) on log(HI) values indicated a statistically significant association between the values. The resulting model was log(NT) titer = 0.0007 + 0.9733 log(HI), with β_1_ *P*‐value <.0001. Separate models for H3N2 and H1N1 were nearly identical, with β_1_=0.9684 (SE=.017) and β_1_=0.9782 (SE=0.0225). When added to the model, age was found to have a statistically significant association with the relation between log(NT) values and log(HI) values. The resulting model, log(NT) = 0.2542 + 0.9663 log(HI) – 0.0055 age (SE_0_=0.079, SE_1_=0.013, SE_2_=0.001), indicates a 0.0055 reduction in log(NT) for every year of age, adjusting for log(HI), yet starting at age 0 with a log(NT) of 0.25 higher than a log(HI).

Hemagglutination inhibition of ≥40 and NT of ≥20 are commonly used as thresholds for indication of immunity, signifying a 50% reduction in the risk of contracting influenza.^5^ Among all of the assays performed, 44.0% and 52.3% measured HI titers ≥40 and NT titers ≥20, respectively (Table 1), indicating, based on these standard thresholds, that there was protective immunity to about half of the influenza strains performed among all study participants. Strain‐specific HI ≥40 results ranged from 0.7% for A/California/07/2009 to 80.8% for A/Fujian/411/2002, and NT ≥20 results ranged from 1.3% for A/California/07/2009 to 84.8% for A/Wuhan/359/1995. Among participants aged <15 years, all (11/11) were found to have at least one HI titer ≥40 and at least one NT titer ≥20, whereas among adults (≥15 years), 2.9% (4/140) had no HI titers ≥40 and only 1/140 had no NT≥20.

We determined HI titer thresholds that predicted NT titer status using multiple titer thresholds and determined which ones maximized sensitivity and specificity to predict NT status. Optimal thresholds were found to vary by serotype and strain. Overall, an HI titer threshold of 20 corresponded to a NT titer of 10, and an HI titer of 40 corresponded to a NT titer of 20 (Table 2). These titer thresholds varied for H3N2 and H1N1 serotypes, with higher titer thresholds for H3N2 (40 and 40) and lower titer thresholds for H1N1 (10 and 20). Strain‐specific HI titer thresholds varied from 10 to 80 corresponding to NT titers of 10 and 20. We observed a mean bias between log(HI) and log(NT) of 0.06 for all strains (Fig. S3). However, the plot of log(NT) versus bias demonstrated a significant negative slope of 0.23 (*P*<.0001), with the greatest magnitude in bias at high NT (Fig. S3).

**Table 2:** Receiver operator characteristic results of comparing NT titers cutoffs with HI titers for all twelve influenza strains, H3N2 strains, and H1N1 strains

	All Strains		H3N2		H1N1	
NT titer cutoff	AUC (95% CI)	HI threshold maximizing sens/spec	AUC (95% CI)	HI threshold maximizing sens/spec	AUC (95% CI)	HI threshold maximizing sens/spec
10	92.8 (91.7–93.9)	20	90.7 (89.1–92.3)	40	93.5 (90.8–96.3)	10
20	93.2 (92.1–94.3)	40	90.8 (89.3–92.3)	40	96.6 (94.6–98.6)	20
40	93.9 (92.9–94.9)	40	92.1 (90.8–93.5)	40	97.6 (96.5–98.8)	20
80	93.5 (92.4–94.6)	40	91.7 (90.3–93.1)	40	97.6 (96.2–98.9)	40
160	94.0 (92.8–95.1)	80	92.3 (90.8–93.8)	80	98.3 (97.3–99.3)	40
320	95.3 (94.2–96.3)	80	93.9 (92.4–95.3)	160	98.9 (98.1–99.7)	80
640	96.8 (95.8–97.8)	160	96.1 (94.8–97.4)	160	98.8 (97.6–99.9)	80

**Figure 4:**
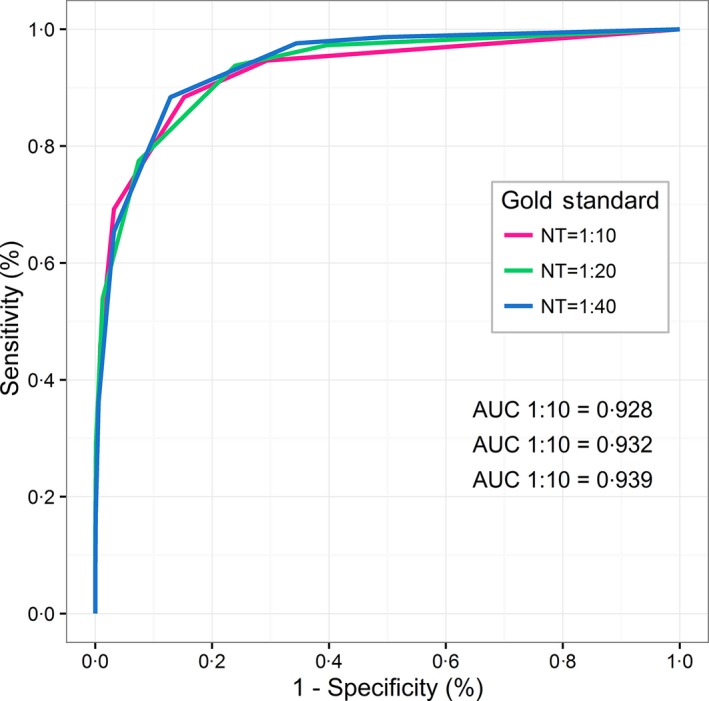
Sensitivity and specificity of predicting NT titer of 10, 20, and 40 using HI data for all influenza strains

Receiver operator characteristic curves comparing results of NT and HI assays indicated similar findings as the correlation tests (Fig. 4/Table 2). Area under the curve (AUC) statistics indicated excellent accuracy of the HI test, as compared with NT as the gold standard, on all titer levels for both overall (all serotypes and years combined) and serotype‐specific analysis (Table 2). Strain‐specific AUCs indicated at least good (>0.80) accuracy for HI tests at NT gold standard titers of 10 and 20 on all strains. A/Shantou/92/2009 (H1N1) and A/California/07/2009 (H1N1) strains, which had the lowest correlation coefficients, produced AUC values of 84.6% and 99.5% for NT titers of 10 and 93.5% and 99.5% for NT titers of 20, respectively.

## 4 Discussion

The primary goal of this study was to gain a better understanding of the direct comparability between hemagglutination inhibition and neutralization assay results for determining level of influenza immunity. Overall, we found that correlation between HI and NT titers was high for all influenza, as well as within serotypes and among most specific influenza strains. While we were unable to determine a consistent equivalence factor between HI and NT titers for influenza, we did confirm previous findings that HI titers were consistent with NT titers.^12^, ^16^ Furthermore, we found age to be significantly associated with the equivalence between HI and NT, with NT more sensitive than HI at young ages, but becoming less sensitive than HI with increasing age. This relationship was also demonstrated by the finding that NT was more sensitive than HI at higher titers, but less sensitive than HI at lower titers. It is unknown whether these relationships are the result of a more rapid decrease in detectible immunity with age by NT as compared to HI, or more simply caused by NT being less sensitive than HI at low titers and more sensitive at higher titers.

The usefulness of a generalizable equivalence factor between NT and HI tests is high, allowing for comparison across studies, better use and understanding of variable levels of immunity, and increased support for use of HI over NT. Although we did not identify a consistent equivalence factor for influenza, we did confirm the findings of previous studies that HI and NT titers were similar within individuals overall. Complicating this comparison is our finding of the significant negative effect of age on the equivalence between HI and NT. While this effect was modest, resulting in a reduction in the equivalent NT titer by about 4% for every 10 years of age, because of the standard use of titer cut points (i.e., 20, 40, 80), this can result in full titer level differences between the two assays. This finding supports previous findings that titer equivalences between HI and NT for adults might not be the same for children.^17^ Furthermore, this points to the potential existence of an underlying biological mechanism of waning immunity or modified immune response as people age that differs between the HI and NT assays. It is not clear whether this effect is truly a factor of biological age or rather a factor of time since initial immunological challenge since these were confounded in our study. However, this finding further exemplifies the challenges of directly comparing HI and NT titer results for understanding influenza immunity. More research is needed to understand these differences by age and how this might impact our understanding of immunity and vaccination.

Our results confirm previous practices of HI titer of 40 corresponding with a gold standard of NT=20 for influenza overall and for H3N2 influenza.^16^ Titer threshold equivalence testing for H1N1 influenza titers, which was determined to be 20 and 20, was limited due to minimal titer results for H1N1 influenza strains. However, strain‐specific HI titer thresholds at which specificity and sensitivity were maximized for gold standard NT ≥20 varied between 20 and 80. As previously found by Stephenson et al.,^12^ producing a single effective equivalence factor, particularly one between an HI titer of 40 and a specific NT titer, was not possible for this study due to dependence on the virus–serum combination and strain‐specific variation, as well as age.

We found that correlation between titers of different strains using the same assay was highest within subtypes and with close proximity in time of virus emergence. These findings indicate multiple possibilities, including the existence of cross‐protective immunity between similar strains, immune response similarity because of antigenically similar strains, or correlation between time of infection and titer level. We included results for both assays as they may characterize strain relationships differently. We are unable to determine which of these or combination thereof is the true cause of this correlation.

An important limitation to determining the equivalence factor in this study was the use of discrete titer thresholds (i.e., 20, 40. 80) and lack of absolute titer data. It is possible, for example, that an equivalence factor found to be 2.0 (NT=20 vs HI=40) could correspond to a true equivalence factor closer to 1.0 if the NT mean absolute titer for this group is 38. Additionally, as our results confirm, we are not able to make any conclusions regarding the comparability or equivalence between HI and NT for A(H1N1)pdm09, as it is unlikely that many of the study subjects had been infected at the time of serum collection. While several patients have low, non‐zero titers, it is possible these were either very recent infections, for which antibodies might not have fully elevated yet, or that there was cross‐reactivity with a different influenza strain. These results are limited to application to seasonal influenza A strains and cannot be generalized to avian influenza, influenza B, or other viruses. Furthermore, with the consistency issues previously observed for NT between laboratories, we cannot guarantee that other laboratories would observe the same consistency between HI and NT titers that we observed. The readout of our assay was based on observing cytopathic effects. Although this has found to be comparable with other assays, some of the variability of our results may be reduced with an automated readout assay.^18^ Finally, the relationship between correlation of titers and age could be driven by the fact that younger individuals had larger titers in general, thus increasing the possibility of larger correlation.

Both tests are widely used to test for immunity to seasonal influenza A, and very few studies have directly compared their results. While NT remains the gold standard, HI offers several advantages that make a better understanding of how it correlates with NT needed. Here, we find overall correspondence between results, but because of the dependence of virus‐serum interactions and an association between age and the HI–NT mapping, no consistent model that can be used across all viruses. With the broad consistency of results between HI and NT, the substantially increase in resources required to conduct NT coupled with the consistency challenges when testing at multiple laboratories, the HI titer might be the more appropriate assay for many studies.

## Competing financial interests

The authors declare no competing financial interests.

## Previous presentation of results

None of these results have been presented previously in any meetings.

## Authors' contributions

ST, JL, and DATC conceived and designed the study; ST, JL, and DATC performed the analysis; HZ, JL, SR, JMR, SW, KOK, YG, CQJ, and DATC contributed reagents/materials/analysis tools; ST and DATC wrote the manuscript; ST, HZ, JL, SR, JMR, SW, KOK, YG, CQJ, and DATC contributed edits and critical review of the manuscript; and HZ, JL, SR, JMR, SW, KOK, YG, CQJ, and DATC designed the empirical study and collected the data and serum samples.
