# Supplementary material for: A comparison of hemagglutination inhibition and neutralization assays for characterizing immunity to seasonal influenza A
Source: Influenza Other Respir Viruses. 2016 Aug 27;10(6):518–24. doi: 10.1111/irv.12408 (PMC5059953; doi:10.1111/irv.12408)
Supplement: Supplementary file 1 [file IRV-10-518-s001.docx]

**Supplemental Figure 1.** ROC showing sensitivity and specificity of predicting NT status using HI titer for different thresholds of NT titer. (a) Results for all strains and multiple NT thresholds of 10, 20, and 40 as outcome, (b) results for H3N2, and (c) results for H1N1.


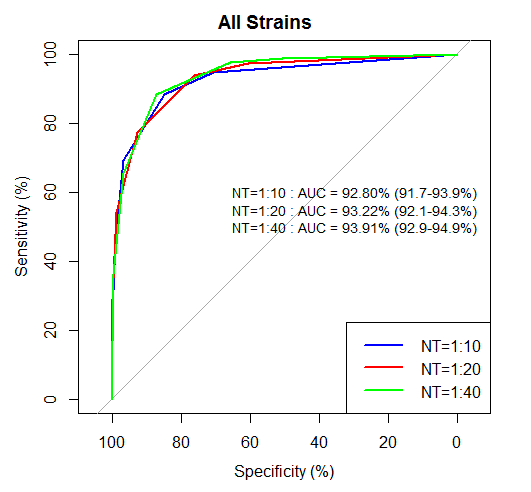

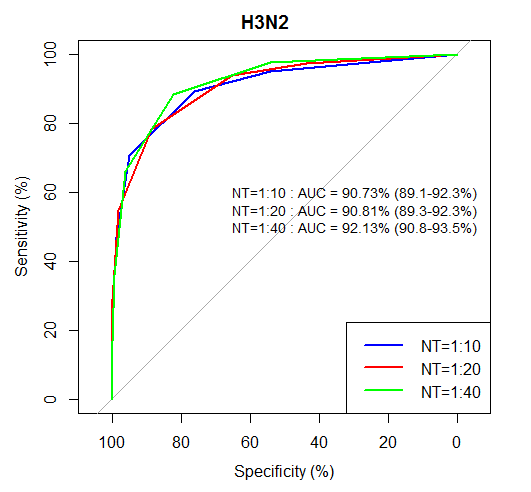


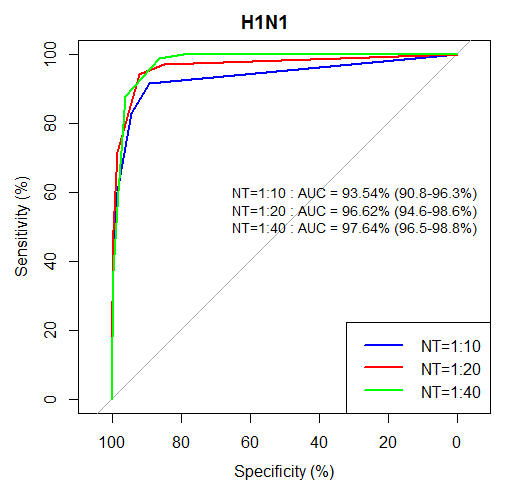


**Supplemental Figure 2.** Cross reactivity correlations by HI and NT. Colors indicate the strength of correlation according to the legend on the right side. The upper right triangle of the matrix is identical to the lower left.


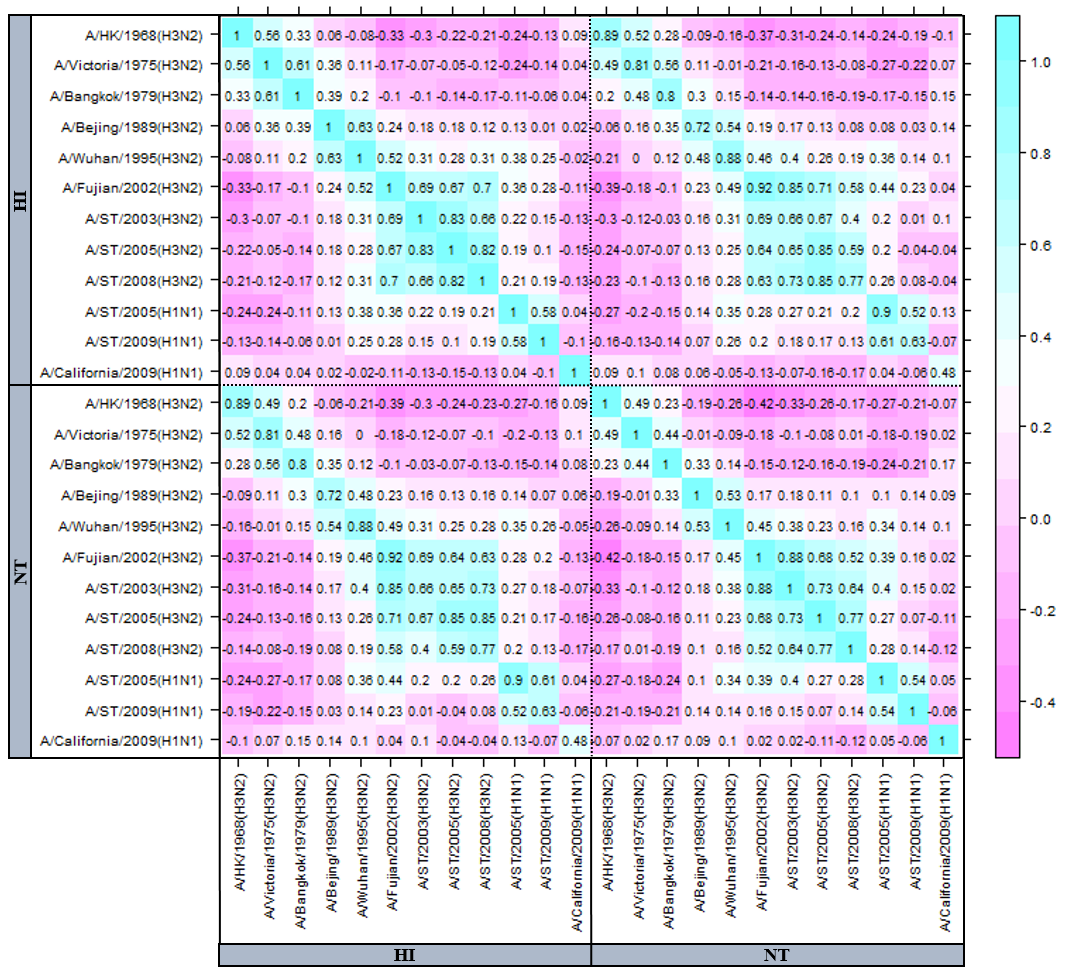


**Supplemental Figure 3.** Bias between NT and HI for (a) all strains, (b) H3N2, and (c) H1N1. Each panel shows a histogram of the bias (log(HI) - log(NT)) for each set of strains, a normal quantile-quantile plot of log(HI) versus log(NT), and a plot of the bias versus log(NT).

1. All Strains


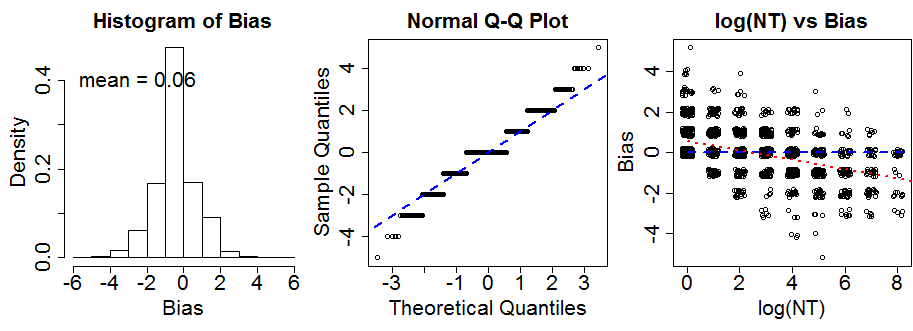


1. H3N2


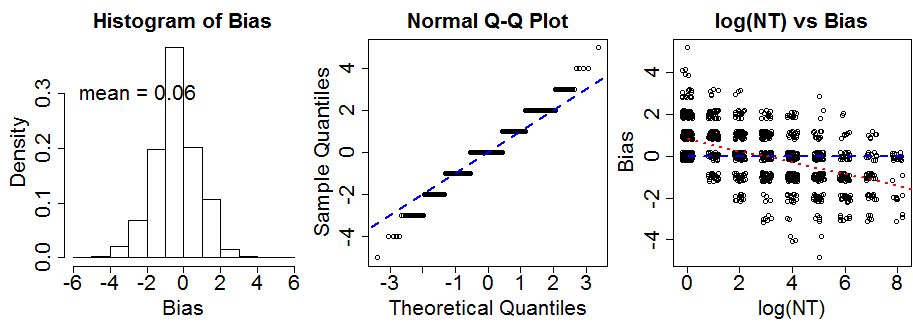


1. H1N1


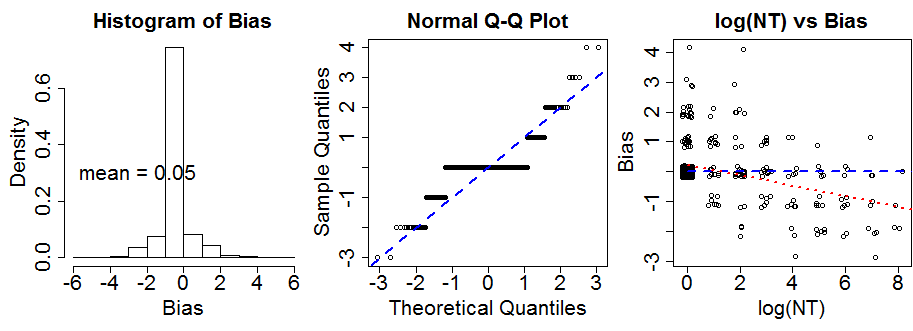


**\**

**Supplemental Table 1.** ROC results comparing NT titer cutoffs of 10 and 20 with HI titers for each individual influenza strain.

|  | NT ≥10 | | NT ≥20 | |
| --- | --- | --- | --- | --- |
| Strain | HI threshold maximizing sens/spec | AUC (95% CI) | HI threshold maximizing sens/spec | AUC (95% CI) |
| A/Hong Kong/1/1968 | 20 | 97.5 (95.5-99.6) | 40 | 95.5 (92.7-98.3) |
| A/Victoria/3/1975 | 20 | 89.6 (84.8-94.5) | 20 | 93.3 (90.0-96.6) |
| A/Bangkok/1/1979 | 40 | 88.2 (81.8-94.7) | 40 | 85.5 (79.2-91.8) |
| A/Beijing/353/1989 | 20 | 84.9 (78.9-91.0) | 40 | 83.5 (77.4-89.6) |
| A/Wuhan/359/1995 | 20 | 92.8 (87.1-98.4) | 40 | 90.0 (83.4-96.5) |
| A/Fujian/411/2002 | 80 | 94.2 (89.6-98.8) | 80 | 94.8 (91.6-97.9) |
| A/Shantou/90/2003 | 40 | 80.6 (71.9-89.3) | 40 | 82.4 (75.2-89.5) |
| A/Shantou/806/2005 | 40 | 93.2 (89.6-96.9) | 40 | 93.2 (89.5-96.8) |
| A/Shantou/904/2008 | 10 | 85.9 (80.4-91.4) | 20 | 91.8 (87.6-95.9) |
| A/Shantou/104/2005 | 20 | 95.2 (92.2-98.3) | 20 | 95.6 (92.7-98.5) |
| A/Shantou/92/2009 | 10 | 84.6 (76.4-92.8) | 20 | 93.5 (86.6-100) |
| A/California/07/2009 | 20 | 99.5 (98.4-100) | 20 | 99.5 (98.4-100) |
